# Examining the impact of genetic testing for type 2 diabetes on health behaviors: study protocol for a randomized controlled trial

**DOI:** 10.1186/1745-6215-13-121

**Published:** 2012-08-01

**Authors:** Corrine I Voils, Cynthia J Coffman, David Edelman, Matthew L Maciejewski, Janet M Grubber, Azita Sadeghpour, Alex Cho, Jamiyla McKenzie, Francoise Blanpain, Maren Scheuner, Margarete Sandelowski, M Patrick Gallagher, Geoffrey S Ginsburg, William S Yancy

**Affiliations:** 1 Center for Health Services Research in Primary Care, Durham Veterans Affairs Medical Center, Durham, NC, USA; 2 Department of Medicine, Duke University Medical Center, Durham, NC, USA; 3 Department of Biostatistics and Bioinformatics, Duke University Medical Center, Durham, NC, USA; 4 Institute for Genome Sciences & Policy, Duke University Medical Center, Durham, NC, USA; 5 Clinical Molecular Diagnostics Laboratory, Duke University Medical Center, Durham, NC, USA; 6 Division of Medical Genetics, VA Greater Los Angeles Healthcare System, Los Angeles, CA, USA; 7 Department of Medicine, David Geffen School of Medicine, Los Angeles, CA, USA; 8 School of Nursing, University of North Carolina at Chapel Hill, Chapel Hill, NC, USA; 9 Veterans Affairs Medical Center (152), 508 Fulton St., Durham, NC, 27705, USA

**Keywords:** Genetic testing, Type II diabetes, Weight loss

## Abstract

**Background:**

We describe the study design, procedures, and development of the risk counseling protocol used in a randomized controlled trial to evaluate the impact of genetic testing for diabetes mellitus (DM) on psychological, health behavior, and clinical outcomes.

**Methods/Design:**

Eligible patients are aged 21 to 65 years with body mass index (BMI) ≥27 kg/m^2^ and no prior diagnosis of DM. At baseline, conventional DM risk factors are assessed, and blood is drawn for possible genetic testing. Participants are randomized to receive conventional risk counseling for DM with eye disease counseling or with genetic test results. The counseling protocol was pilot tested to identify an acceptable graphical format for conveying risk estimates and match the length of the eye disease to genetic counseling. Risk estimates are presented with a vertical bar graph denoting risk level with colors and descriptors. After receiving either genetic counseling regarding risk for DM or control counseling on eye disease, brief lifestyle counseling for prevention of DM is provided to all participants.

**Discussion:**

A standardized risk counseling protocol is being used in a randomized trial of 600 participants. Results of this trial will inform policy about whether risk counseling should include genetic counseling.

**Trial registration:**

ClinicalTrials.gov Identifier NCT01060540

## Background

Genetic testing has become an increasingly viable option for conveying the risk of developing complex chronic diseases [1]. Genetic testing for this purpose currently is not a routine part of primary care. Barriers may include a lack of resources for testing, a lack of genetic counselors to deliver the results, and uncertainty about whether knowing genetic risk prompts risk reduction behaviors [2]. Evidence of the clinical utility of providing genetic testing results is needed to inform policy regarding the use of genetic testing for complex chronic diseases in clinical care.

Diabetes mellitus (DM) is an ideal prototype disease for examining this issue for several reasons. First, DM is a highly prevalent and costly chronic disease that results in debilitating microvascular and macrovascular complications and impaired quality of life [3,4]. Second, genetic abnormalities in complex chronic diseases such as DM involve multiple genes such that testing determines disease risk [5]. This is in contrast to Mendelian disorders, which result from a mutation in a single causative gene and for which single-gene tests determine, definitively, the presence or absence of disease, regardless of lifestyle choices. Third, much is known about the environmental and genetic contributors to the development of DM, and the evidence suggests that behavior change is the best way to prevent its development, even when gene polymorphisms are present [6].

We are conducting a randomized controlled trial (RCT) to examine the comparative effectiveness of risk counseling using conventional risk factors for DM versus counseling that additionally includes genetic testing results. In this article, we report on the study design, procedures, and development of the counseling protocol.

## Methods/Design

### Study design and overview

**Figure 1:**
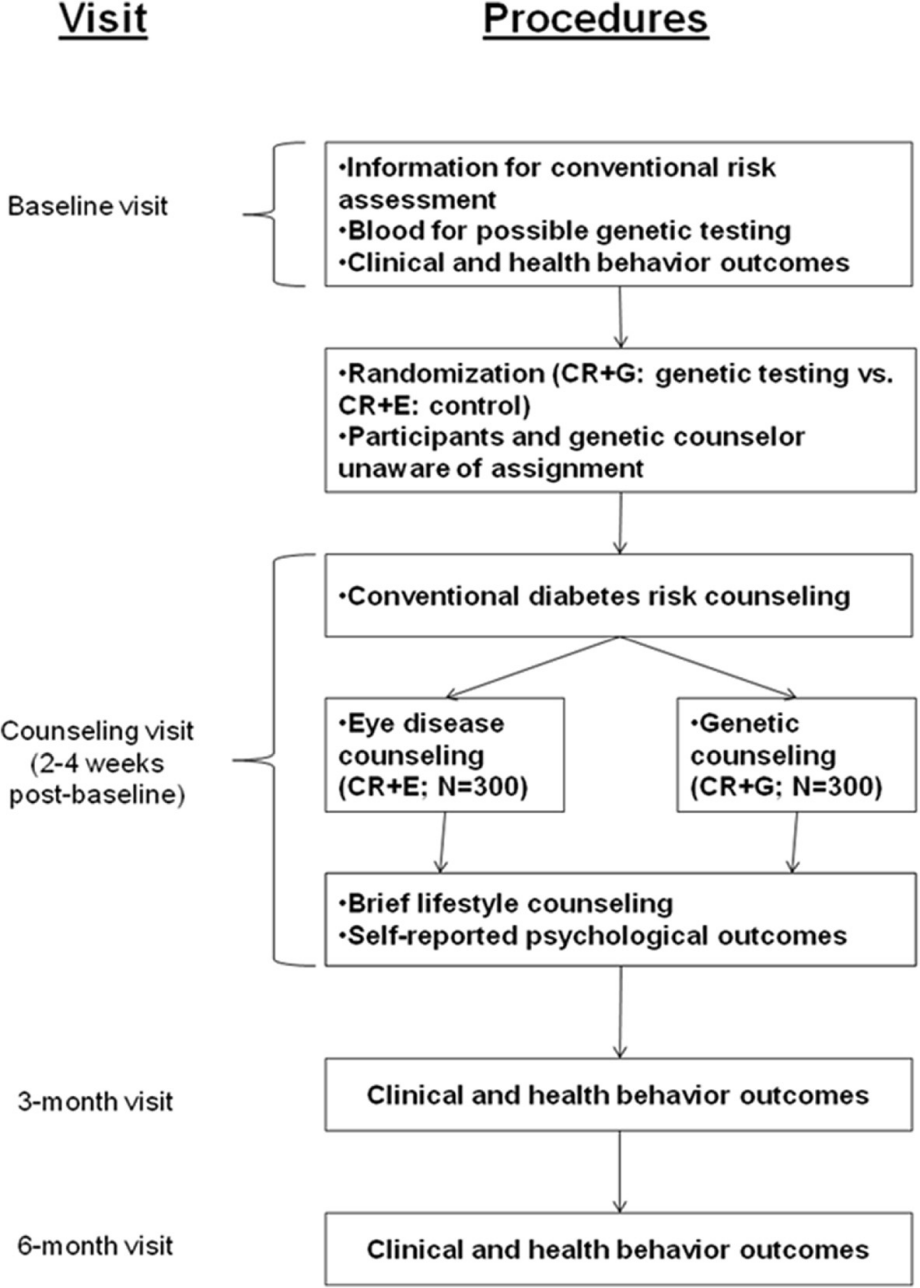
Study overview.

The ongoing study is a two-arm RCT to determine the effect of genetic testing for DM on clinical outcomes and health behaviors. At baseline, participants provide written informed consent, information for conventional risk assessment, blood samples for fasting plasma glucose (FPG) and possible genetic testing, and baseline clinical and health behavior outcomes (Figure 1). Eligible participants are then randomized to receive conventional risk plus genetic test result counseling (CR + G) or conventional risk plus control eye disease counseling (CR + E), stratified by family history (unknown/low vs. moderate/high) and body mass index (BMI; < 35 vs. ≥ 35 kg/m^2^). Two to 4 weeks following baseline, all participants return for a risk counseling visit with a genetic counselor that does (CR + G) or does not (CR + E) include delivery of genetic test results and associated counseling. Psychological outcomes are assessed immediately following counseling and at 3 and 6 months. Behavioral and clinical outcomes are assessed at 3 and 6 months, with 3 months as the primary endpoint.

### Study population and recruitment

Participants are recruited from the Durham Veterans Affairs Medical Center (VAMC) and satellite clinics. Ethical approval was obtained from the Institutional Review Board and Research and Development committees at the Durham VAMC. Eligibility is confirmed by an electronic data pull followed by a screening telephone call and a baseline visit (see Table 1 for eligibility criteria). A recruitment letter is mailed to patients meeting inclusion criteria who have a clinical appointment in the next 3 weeks. If patients do not opt out of the study by calling a toll-free number, a research assistant (RA) contacts them by phone within 2 weeks to describe the study, assess interest, and administer a cognitive screen [7]. Patients may also self-refer in response to flyers posted in the medical center. Interested and eligible patients are then scheduled for the baseline study visit. Patients receive reminder letters with fasting instructions 1 week before their scheduled appointments and reminder phone calls the day before their appointments.

**Table 1:** Eligibility criteria and method of ascertainment

	**Criterion**	**Method**
Inclusion criteria	Age 21–65 years	Electronic data pull
	Body mass index ≥ 27 kg/m^2^	Electronic data pull (≥ 26 kg/m^2^ to allow for error), screening telephone call, and confirmed at baseline visit
Exclusion criteria	Diagnosis of diabetes mellitus	Electronic data pull, confirmed in telephone call
	Fasting plasma glucose ≤ 125 mg/dl on more than one occasion	Electronic data pull
	Hemoglobin A1c >7%	Electronic data pull
	Taking diabetes medication	Electronic data pull, confirmed in telephone call
	Intentional weight loss of at least 5 lbs in previous 3 months	Screening telephone call
	Enrolled in a research study or any program focused on lifestyle changes	Screening telephone call
	Unable to provide informed consent or answer survey questions unassisted	Screening telephone call
	Residing in nursing home or receiving home health care	Screening telephone call
	At least 1 error on a validated 6-item screen for cognitive impairment [7]	Screening telephone call
	Fasting plasma glucose ≤ 125 mg/dl at baseline	Baseline visit

### Baseline visit

At the baseline visit, an RA describes the study, reviews the risks and benefits of genetic testing, and reviews the randomization process. After any questions are addressed, participants provide written informed consent. Participants are asked not to obtain genetic testing from outside resources while enrolled in our study. Age, sex, race, family history, weight, and height are collected for use in calculating cumulative lifetime risk for DM. History of DM is obtained for first- and second-degree relatives. Upon confirmation that patients have fasted for 12 h, blood is drawn for FPG, fasting insulin, and possible genetic testing. Participants complete a measure of numeracy [8], then the RA administers all other measures orally. Participants receive $25 for this 1-h visit. Final study eligibility is determined based on the baseline FPG results, usually available within 24 h.

### Randomization

Participants are randomized to the CR + G versus the CR + E arms in blocks within four strata defined by weight status (BMI <35 kg/m^2^ vs. ≥ 35 kg/m^2^) * family history of DM (unknown/low vs. moderate/high). We wanted to ensure balance in each arm on BMI status as weight trajectories, and adherence to recommendations may differ depending on severity of obesity. We also wanted to ensure balance on family history of DM in each arm as individuals with a strong family history may be more likely to engage in preventive lifestyle changes [9]. The project coordinator enters the values of the stratification variables and FPG into the study database, and the randomly assigned study arm is returned for eligible patients. Then, due to budget constraints, only blood samples for CR + G participants are sent for genetic testing.

Only the project coordinator and Master’s-level statistician have access to the randomization section of the study database. RAs are blind to arm assignment, and arm assignment is not revealed to participants or the genetic counselor until during the counseling session, after the conventional risk counseling has been delivered. Because randomization occurs prior to the genetic counseling session (to determine which blood samples will be extracted for genetic testing), participants are considered randomized whether or not they attend the counseling appointment and will be analyzed in an intent-to-treat manner.

### Genetic testing

To date, single nucleotide polymorphisms (SNPs) that have been associated with DM are weakly associated, typically yielding odds ratios (OR) <2.0, although combinations of multiple SNPs have resulted in larger ORs [10]. We are examining how genetic information influences health behaviors and hypothesize that even markers with small ORs may affect health behaviors. We selected 3 DM-related genes (TCF7L2, PPARg, and KCNJ11) in which to test SNPs because, when we designed this study, they were among the most studied SNPs, even in populations of varying ethnicities [10].

The Duke Clinical Molecular Diagnostics Laboratory is performing the genetic testing. The regions of the KCNJ11, PPARG, and TCF7L2 genes, which encompass the desired single nucleotide polymorphisms of interest [Rs5219T > C, Rs1801282C > G, Rs7903146C > T, respectively], are amplified using three primer pairs. Purified genomic DNA is used for polymerase chain reaction (PCR). The primers used in the PCR reactions contain M13 universal primer “tails” at their 5′ ends and have 3′ ends that are homologous to their genomic target sequence. The resulting PCR products are treated with an exonuclease/ phosphatase mixture (ExoSAP-IT: USB Corporation) to remove excess PCR primers and nucleotides. These purified DNA amplicons are then sequenced using universal M13 forward and reverse primers [M13 Forward (17 bp) and M13 Reverse (17 bp)] and the Big Dye Terminator v3.1 Cycle Sequencing Kit (Applied Biosystems). These products are then purified with the Big Dye XTerminator Purification Kit (Applied Biosystems) and resolved using the ABI 3130xl Genetic Analyzer. Data are analyzed using the ABI Data Collection software v3.0, Sequencing Analysis software v5.2 and SeqScape software v2.5. Sequences are compared to the reference DNA sequence (GenBank accession: K:NT_009237.18, P:NT_022517.18, T:NT_030059.12).

### Risk stratification

We provide patients with risk estimates for up to four different DM risk factors (lifetime risk based on age, sex, race, and BMI; risk based on family history; risk based on FPG level; and, for those randomized to the CR + G group, risk based on genetic testing for 3 DM-related genes) rather than one global DM risk value because there is no well-validated algorithm that incorporates various DM risk factors into a single prediction score similar to the Framingham risk score for cardiovascular disease risk. We categorize participants’ risk into three levels based on validated algorithms (low, moderate, or high; Table 2) to provide patients with a means of “fairly” comparing their risk across the different measures given the fact that the reference groups for the numerical risk categories often differ (e.g., lifetime DM risk based on BMI, race, sex, and age being presented as a percentage versus DM risk based on FPG being presented as an odds of developing DM in the next 5 years). Furthermore, numerical risk information tends to be poorly understood by patients [11], particularly when information is unfamiliar and presented in isolation [12,13]. Rather, people tend to focus more on the gist of risk information [14].

**Table 2:** Low, moderate, and high-risk values for each risk category

**Risk category**	**Low risk**	**Moderate risk**	**High risk**
**population-based risk**	**<20%**	**20-40%**	**>40%**
Family history	Only (1) one second-degree relative with DM from one or both sides of family; or no family history of DM	Only (1) one first- and one second-degree relative with DM from same lineage; (2) one first-degree relative with DM; (3) mother and father with DM; or (4) two second-degree relatives from same lineage with DM	At least (1) two first-degree relatives with DM from same lineage; (2) one first- and two second-degree relatives with DM from same lineage; or (3) three second-degree relatives with DM from same lineage
Fasting plasma glucose (FPG)	<100 mg/dl	100-109 mg/dl	110-125 mg/dl*
Copies of high-risk alleles from TCF7L2, PPARg, KNCJ11 (possible range 0–6)	0-2	3	4-6

* Participants with FPG >125 mg/dl are excluded.

Population-based risk estimates are taken from lifetime risk tables based on age, sex, race, and BMI [15]. We classified <20% lifetime risk as low, 21-40% as moderate, and >40% as high. Family history of DM is based on first- and second-degree relatives, and classification into risk levels is based on a published algorithm [16]. Although the American Diabetes Association guidelines consider prediabetes present when FPG >100-125 mg/dl, analyses indicate a high false-positive rate for values between 100 and 109 mg/dl [17]. Therefore, participants are classified according to whether their FPG is <100 mg/dl (low), 100–109 mg/dl (moderate), or 110–125 mg/dl (high). Participants with baseline FPG >125 mg/dl become ineligible and are encouraged to follow-up with their primary care provider. Based on the distribution of the possible combinations of the high-risk alleles that we are testing in a previous study [10], we designated 0–2 high-risk alleles as low risk, 3 as moderate, and 4–6 as high risk.

### Development of risk counseling protocol

#### Conventional risk counseling (both arms)

All counseling in both arms is delivered by the same genetic counselor to avoid the possibility that differences between arms could be attributed to the differential training if another individual delivered the risk counseling in the CR + E arm. One genetic counselor (AS) performed the counseling for the first 342 randomized participants, and a second genetic counselor is performing the genetic counseling for the remaining randomized participants. Analyses will explore effects by counselor.

The counseling protocol comprises a flipbook with written and graphical information accompanied by an oral presentation. The session begins with a definition of DM and its prevalence, possible negative outcomes, and risk factors. The counselor briefly mentions that genes are involved in the development of DM and emphasizes that, although family history incorporates genetic information, family history also reflects shared lifestyle and environment. Next, the counselor provides participants with personalized risk estimates for lifetime risk, family history, and FPG.

**Figure 2:**
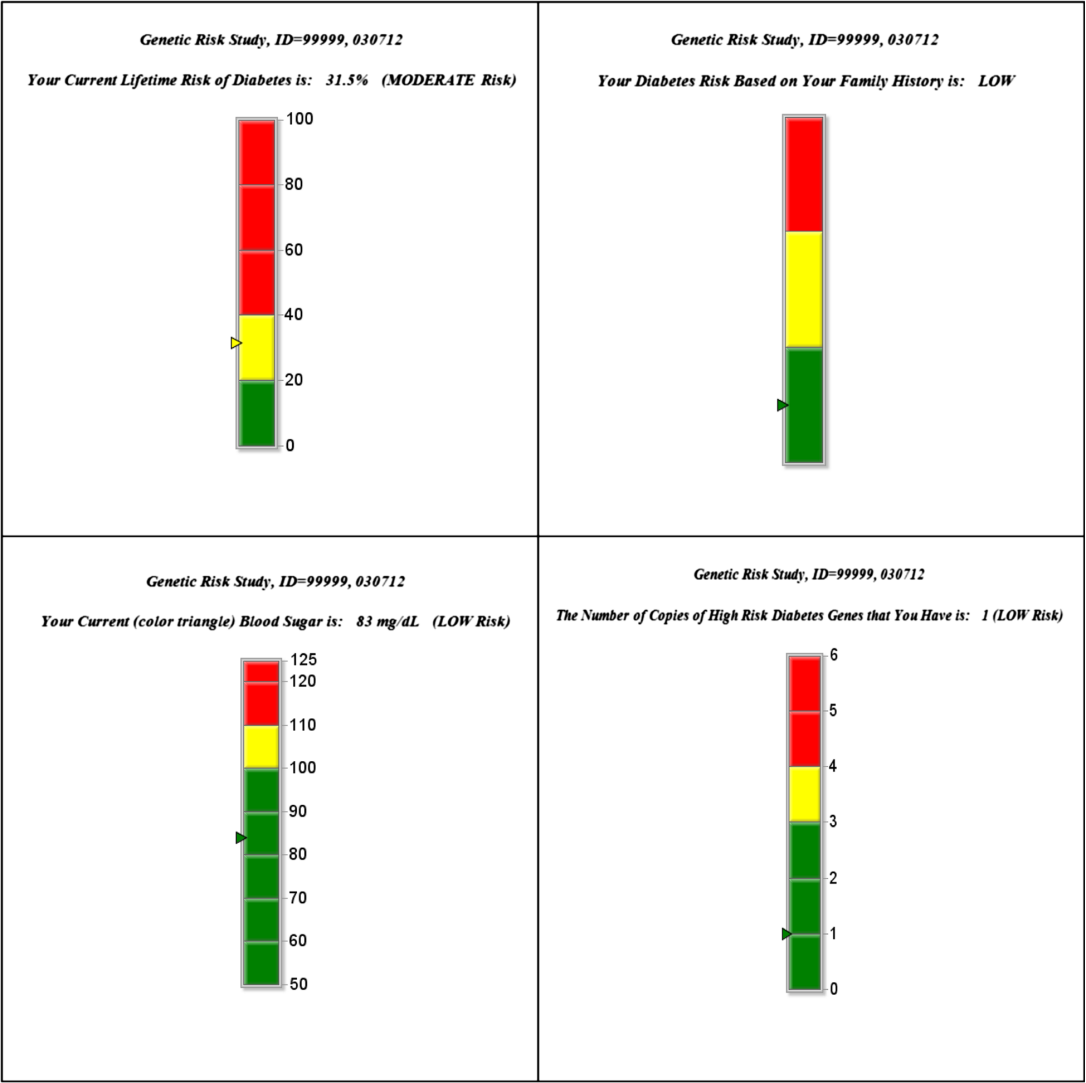
Sample graphs for lifetime risk, family history, fasting plasma glucose, and genetic testing.

To evaluate different ways of presenting personalized risk information, we pilot tested the counseling protocol in 25 patients meeting eligibility criteria. In a previous study, a vertical bar graph and a thermometer, accompanied by color, were preferred methods of conveying cardiovascular risk level by providers and patients [18]. Therefore, in our pilot study, we presented risk levels in a vertical thermometer format with low risk level colored green, moderate colored yellow, and high colored red. A colored arrowhead denotes the risk level positioned on the thermometer graph at the value of the risk factor (Figure 2).

At the end of the counseling session, CIV conducted qualitative interviews to examine participants’ interpretation of the information, solicit feedback on the counseling protocol, and evaluate different pictorial presentations of personalized risk information. Specifically, participants viewed a pie graph, a vertical bar graph, a horizontal bar graph, and a speedometer, all in black and white and in color versions (low risk = green, moderate risk = yellow, high risk = red). Participants were asked to compare the graphs, indicate which best conveyed risk level, and for their interpretation of the descriptors and colors. Participants preferred the vertical thermometer, indicated that the colors aided in interpretation of the risk level, and indicated that the colors matched the descriptors.

#### Genetic risk (CR + G)

The genetic counselor provides a brief review of genetics and indicates that studies have linked several genes to the development of DM. The genetic counselor further indicates that genetic testing for DM does not yield definitive risk estimates, but rather that it supplements the risk information already considered with the other three risk categories. Next, the genetic counselor provides participants with their personal genetic testing results in a manner similar to the other risk estimates using a vertical thermometer bar graph (last graph in Figure 2). The genetic counselor emphasizes that we tested only three of several genes that are associated with increased DM risk and that lifestyle modification can prevent or delay the onset of DM even if genetic results indicate an increased risk.

#### Control counseling (CR + E)

To ensure that the control arm (CR + E) receives equal duration of genetic counselor contact, information is reviewed for age-related macular degeneration, cataracts, and glaucoma. We chose this control topic because the lifestyle modifications recommended to prevent these eye diseases overlap little with those recommended to prevent DM. Additionally, although DM is a risk factor for eye disease, we chose eye disease topics that would not emphasize this risk so that the information presented would be unlikely to add to control participants’ motivation to prevent DM. Participants receive information about risk factors, screening, and behaviors to reduce their risk for the three eye diseases, although no individualized risk information is presented. Ten pilot participants completed a risk counseling session with eye counseling instead of genetic counseling to ensure that participants understood the information and to match the duration of the eye disease counseling to the duration of the genetic counseling (approximately 10 min). Thus, any difference between arms could not be attributed to the increased amount of time spent with participants in the CR + G arm, but rather the content of the genetic counseling.

#### Lifestyle counseling (both arms)

Following genetic or eye disease counseling, the genetic counselor engages participants in brief lifestyle counseling. The counselor informs participants that lifestyle changes can help ameliorate risk, even in the presence of family history or genetic abnormalities. Using a semi-structured script that operationalizes some basic principles of motivational interviewing, the genetic counselor encourages participants to set physical activity and/or dietary intake goals, with an emphasis on simple, short-term, and measurable goals [6]. Goals are recorded on a sheet to take home, and participants are encouraged to review their progress monthly and adjust goals accordingly. Participants are provided with a summary statement containing their risk information, including genetic testing results if in the CR + G arm; the National Institutes of Health *Game Plan* resources, which are based on the Diabetes Prevention Program and provide comprehensive information about DM and tools for adopting prevention behaviors; an informational pamphlet on DM that we created; and a list of resources available at VA, including website addresses. Participants in the CR + G arm also receive the NHGRI publication *A Guide to Your Genome* to aid in genome science education and comprehension of genetic information. Participants receive $25 for this visit, which can take up to 1.5 h.

### Measures

Demographics are assessed at baseline by an RA. Psychological outcomes are assessed by the genetic counselor immediately following the risk counseling. The remaining outcomes are assessed by a blinded RA at 3 and 6 months, with 3 months as the primary endpoint. Participants receive $25 for the 3- and 6-month outcome visits.

#### Primary outcome

The primary outcome is weight because it is sensitive to lifestyle modifications [19] and is highly correlated within an individual over time, even in studies of intensive weight loss interventions [20]. Weight change was more influential than physical activity level or dietary fat intake for the prevention of DM in the Diabetes Prevention Program [21]. Body weight is measured on a standardized digital scale, with participants wearing light clothing and with accessories and shoes removed.

#### Psychological outcomes

Likelihood is assessed with the item, “What are your chances of getting type 2 diabetes in your lifetime?” (1 = *definitely will not get diabetes* to 7 = *definitely will get diabetes)*. The Brief Illness Perception Questionnaire measures dimensions of illness captured by the Common Sense Model, including consequences, timeline, personal control, treatment control, identity, concern, understanding, emotional response, and causal attributions [22]; items are modified to focus on developing DM in the future as opposed to now. Readiness to change diet and increase physical activity is assessed with single items [23]. Self-efficacy to follow a diet is assessed with the revised Eating Self-Efficacy Scale [24]. Self-efficacy to engage in physical activity is assessed with the Self-Efficacy for Exercise scale [25]. Intentions to modify dietary intake and increase physical activity are assessed with seven semantic differential items.

#### Reactions to genetic testing

The Multidimensional Impact of Cancer Risk Assessment Questionnaire is administered in the CR + G arm only at 3 and 6 months to assess the psychosocial impact of receiving a genetic test result and was adapted for DM [26].

#### FPG, fasting insulin, and homeostasis model assessment of insulin resistance (HOMA-IR)

Improvements in insulin action are assessed by measurement of FPG and insulin levels, with subsequent calculation of HOMA-IR. Hemoglobin A_1c_ was not measured because it was not recommended for diagnosis of DM when the study began.

#### Dietary intake

Dietary intake is assessed with the Block Brief 2000 Food Frequency Questionnaire, which includes color photos representing portion sizes to aid participants in estimating typical portion size, and instructions ask participants to check off foods they have eaten during the past 3 months, indicating portion size and how often they eat each item.

#### Physical activity

Daily physical activity is assessed by the long version of the International Physical Activity Questionnaire (IPAQ) [27], which assesses activity over the past 7 days in the domains of occupation, transportation, yard/garden, household, leisure, and sitting. The IPAQ provides estimates of metabolic equivalent tasks energy expenditure, which can be reported for each activity or as a total score.

#### Utilization of weight loss resources

Participants rate how frequently they have used the following resources since their risk counseling session (*never, rarely, sometimes,* and *often*): written materials provided during the counseling session; MyHealtheVet, a website for veterans that offers health resources; other websites; home exercise equipment; books related to lifestyle changes; medication to prevent DM; weight loss medication; weight loss surgery; the VA’s MOVE! weight loss program; a personal trainer; a nutritionist; a weight loss program; recreation center or fitness facility; park or greenway; and physician-recommended regimen.

### Primary analysis

Primary analyses will be conducted on an intent-to-treat basis; participants will be analyzed in the group to which they were assigned, regardless of whether they attend the counseling session, using all available data [28]. Statistical analyses will be performed using SAS for Windows (Version 9.2: SAS Institute, Cary, NC) and R (http://www.R-project.org).

Our primary hypothesis is that mean weight loss in the CR + G group will be at least 6 lbs greater than in the CR + E group after 3 months. Weight is a continuous variable, and we will fit longitudinal models examining the short-term effects of the intervention using linear mixed models [29], where the baseline and 3-month weight measurements will be part of the outcome vector. This method handles dropouts in a principled manner. Depending on the type and scope of missing data, we will also explore multiple imputation as a strategy to use in conjunction with our primary analytic tools [30].

### Secondary analyses

The secondary hypotheses of this study are that mean perceived risk in the CR + G group will be greater than in the CR + E group immediately following counseling; that mean improvements in physical activity, caloric intake, and insulin resistance in the CR + G group will be greater than in the CR + E group after 3 months; and that mean improvements in weight loss, physical activity, caloric intake, and insulin resistance in the CR + G group will be greater than in the CR + E group after 6 months. Because all secondary outcomes are continuous, we will use the same analysis plan as for the primary outcome analysis. Hypotheses concerning perceived risk and the 3-month outcomes will be tested by the coefficient for the treatment × time interaction. For the hypothesis concerning 6-month outcomes, the models will include an additional time point. We will examine the treatment × time interaction, and, using contrasts among the model parameters, we will estimate the between-group differences after 3 and 6 months of follow-up. We expect the effect of the genetic testing to be largest at the 3-month time point because it is in closer proximity to the counseling session. A 6-month between-group difference that remains significant and is of similar magnitude to the 3-month difference would imply that the intervention effect was sustained.

### Power and sample size considerations

We plan to enroll 300 participants in each arm of the study (total n = 600). Our power calculation is based on our primary outcome of weight, under the null hypothesis that there will be no between-group difference in weight at the 3-month time point. The sample size is based on methods appropriate for analysis of covariance analyses in randomized trials [31], where we applied a two-sample *t*-test sample size calculation for the between-group difference at the 3-month time point multiplied by a factor 1-(*rho*)^2^, where *rho* represents the Pearson correlation between baseline and 3-month time point outcome measures.

Based on our prior work [32], we estimated a standard deviation of approximately 55 lbs at the 3-month time point and a correlation of 0.90 between baseline and 3-month weights. Using these variability and correlation estimates, and assuming an attrition rate of 10% at 3 months with an α = 0.05 (two-sided) and sample size of 300 per group, we will have approximately 80% power to detect a 6-lb difference in weight between the CR + G and CR + E groups. A 6-lb difference is considered clinically meaningful [33] based on evidence from the Finnish Diabetes Prevention Study, in which a net weight loss of 7.5 lbs at 1 year reduced risk for DM by 58%.

### Cost-effectiveness analysis

To assess the cost-effectiveness of the intervention, we will examine variation in health-care and intervention costs between CR + G and CR + E arms, and variation in effectiveness between arms, to calculate an incremental cost-effectiveness ratio that summarizes the relative costs and benefits of the genetic counselor-led intervention. The effectiveness measure will be weight (in pounds). To conduct the cost-effectiveness analysis from a “limited” social perspective [34], several additional costs will be collected beyond health-care costs, including intervention costs incurred by study staff and patients, patient out-of-pocket and travel costs, and indirect costs incurred by patients because of loss of productivity.

To capture the amount of time the genetic counselor spends with each patient and the total amount of time spent documenting interactions with the patients, and to differentiate these intervention activities from research activities, the genetic counselor logs these time commitments by patient and date. Intervention costs attributable to the RA, project coordinator, study investigators, and genetic counselor will be based on the specific personnel’s annual salary plus benefits. Costs for intervention supplies (computers, office furniture, and telephones) will be based on their acquisition price from the manufacturer, and office space will be calculated based on standard VA rates and will be allocated over their expected lifetime of use. Patient time costs will be based on hourly wages calculated from Bureau of Labor Statistics data and on the average amount of time spent in the intervention, not counting outcome assessments.

## Discussion

Incorporating genetic testing for common, complex chronic diseases such as DM requires resources not commonly found in primary care settings, including genetic testing capabilities and genetic counselors. We developed a risk counseling protocol for conventional DM risk factors and the addition of genetic testing results, which is being evaluated in a randomized trial of 600 participants. The results of the trial will contribute to the evidence base of the clinical utility of genetic information for motivating patient behavior change.

### Limitations

One possible limitation of this study is that, despite many patients’ enthusiasm for genetic testing, as documented in the existing literature and in our pilot study of veterans, some patients may be reticent to undergo genetic testing. Therefore, our recruitment rate may be lower than for studies not using genetic testing. Yet, given the large number of patients (>5,700) at the Durham VAMC who are aged 21–65, BMI ≥ 27 kg/m^2^, with no prior diagnosis of DM, we should have more than adequate numbers from which to recruit.

Another limitation is the relatively low intensity of the genetic counseling. Sensitive to the fact that we are not testing a lifestyle change intervention per se, but the effect of additional, specific knowledge of genetic risk factors, we purposefully refrained from using an intensive patient intervention, relying instead upon the measurement of behaviors and sensitive clinical outcomes (weight, FPG, insulin) and a large sample size to compensate for the possibility that observed effect sizes will be small. If the genetic counseling shows promise for motivating lifestyle behavior change, then pairing it with a counseling program may ultimately enhance its effectiveness.

Additionally, we are using only three of several genes that have been associated with DM for the genetic component of the risk counseling. We chose these three genes based on their strength and consistency of association among numerous studies and populations, recognizing that including additional genes might improve the accuracy of the risk information marginally. The number of genes associated with DM increases over time so that additional associations will be found as this study is conducted. The purpose of this study is to evaluate the behavioral impact of genetic risk information rather than to optimize the accuracy of the information.

Finally, due to the relatively short duration of the study, weight is used as a surrogate for the incidence of DM. Future studies may examine whether genetic testing prevents or delays the onset of DM.

### Strengths

The current study will address limitations of previous studies and advance our knowledge about the role of genetic testing for a chronic disease in the following ways. First, the proposed study will be among the first to determine whether genetic risk counseling for DM results in significant changes in health behaviors and clinical outcomes.

Another strength is that the RCT design will allow us to assess whether any changes in health protective behaviors occur because of the addition of genetic counseling to conventional risk counseling. Most studies of the effect of genetic counseling have been observational, limiting the conclusions that can be drawn [35].

Finally, a genetic counselor delivers the genetic results, representing the best-case scenario to determine the efficacy of genetic risk counseling. If this study proves positive, then we will have developed a model that could perhaps be expanded using other types of providers, who would be more likely to use genetic testing in primary care (e.g., internists, nurse practitioners, physician assistants).

## Conclusion

A standardized protocol for delivering risk reduction counseling to patients at risk for developing DM has been developed and could be incorporated into primary care. Results of the trial will inform policy about whether this counseling should additionally include genetic testing results. Genetic testing is becoming more widespread, appearing more in clinical settings and now offered as a direct-to-consumer service that can be purchased from a website and performed simply by mailing in a saliva sample [1,36]. Novel diagnostic tests are sometimes integrated into practice before understanding their full impact on diagnostic and treatment decisions, or carefully weighing their physical, mental, and financial costs. Like diagnostic tests that predict the presence of disease, genetic risk information should be evaluated not only for accuracy, but also for integration into clinical care and the ability to affect treatment decisions and behaviors. Results from this trial will contribute to the evidence base to inform the future consumption of genetic testing for complex chronic diseases.

## Trial status

Enrollment for the RCT phase of this project began in January 2011 and will be completed in August of 2012.

## Abbreviations

BMI, Body mass index; CR + E, Conventional risk + eye disease counseling; CR + G, Conventional risk + genetic counseling; DM, Type 2 diabetes mellitus; DNA, Deoxyribonucleic acid; IPAQ, International Physical Activity Questionnaire; FPG, Fasting plasma glucose; OR, Odds ratio; PCR, Polymerase chain reaction; RA, Research assistant; RCT, Randomized controlled trial; SNP, Single nucleotide polymorphism; VA, Veterans Affairs; VAMC, Veterans Affairs Medical Center.

## Competing interests

The authors declare that they have no competing interests.

## Authors’ contributions

CIV obtained funding, participated in the design and coordination, assisted in refining the risk communication graphs, and drafted the manuscript. CJC participated in the design, assisted in refining the risk communication graphs, performed the power analysis, designed the analytic plan, and assisted in drafting the manuscript. DE participated in the design, assisted in refining the risk communication graphs, developed procedures for the rapid enrollment of our large study population, and revised the manuscript critically for important intellectual content. MLM developed the cost evaluation, assisted in drafting the manuscript, and revised it critically for important intellectual content. JMG assisted in developing the study database, wrote the code for generating the risk communication graphs, and assisted in drafting the manuscript. AS created the genetic counseling protocol and revised the manuscript critically. AC made substantial contributions to conception and design of this project. JM made substantial contributions to the acquisition of data and assisted in developing the study database. FB designed the DNA extraction and SNP testing protocol. M Scheuner made substantial contributions to conception and design of this project. M Sandelowski made substantial contributions to the qualitative pilot study. PG made substantial contributions to conception and design of this project. GG helped develop the study consent process and method of delivery of genetic testing results. WSY obtained funding for the project, assisted in drafting the manuscript, and revised it critically for important intellectual content. All authors read and approved the final manuscript.
